# Response to “Evaluation of the LMUP in Ethiopia: Requirements, challenges and best practice”

**DOI:** 10.1016/j.conx.2023.100099

**Published:** 2023-08-11

**Authors:** Celia Karp, Caroline Moreau, Solomon Shiferaw, Assefa Seme, Mahari Yihdego, Linnea Zimmerman

**Affiliations:** Department of Population, Family and Reproductive Health, Johns Hopkins Bloomberg School of Public Health, United States; Department of Population, Family and Reproductive Health, Johns Hopkins Bloomberg School of Public Health, United States; Soins et Santé Primaire, CESP Centre for Research in Epidemiology and Population Health U1018, Inserm, F-94805 Villejuif, France; Department of Reproductive Health and Health Service Management, School of Public Health, Addis Ababa University, Ethiopia; Department of Population, Family and Reproductive Health, Johns Hopkins Bloomberg School of Public Health, United States

To the Editor:

We thank the authors for their thoughtful response to our paper, entitled “Evaluation of the London Measure of Unplanned Pregnancy (LMUP) among a nationally representative sample of pregnant and postpartum women Ethiopia” [1]. We appreciate the authors’ dedication to improving measurement of pregnancy preferences and share in this goal.

The purpose of our study was to use the LMUP, as recommended by its authors [2], and understand how the measure worked in Ethiopia. We found that in this nationally representative setting, two of the LMUP items reflecting pre-pregnancy behaviors (i.e., preconception care, contraceptive use) did not perform well, evidenced by poor correlation with other LMUP items and low principal components analysis (PCA) loadings. We address how these item-specific results contradict those of another facility-based study in Ethiopia [3] and align with similar challenges in other settings, such as Sierra Leone [4], India [5], and Malawi [6]. Our psychometric approach for evaluating the LMUP in Ethiopia mirrors that of other researchers who have tested the measure in low-resource contexts (i.e., classical test theory with principal components analysis, hypothesis testing, and confirmatory factor analysis). We expanded this approach through sensitivity analyses that examined abbreviated versions of the measure, based on observed item properties and challenges in Ethiopia. Our intention with this part of the analysis was to maximize measure performance with the minimum number of items. While our recommendation is that “researchers and programs using the LMUP in Ethiopia and similar settings with limited preconception care and contraceptive use may consider removing the two pregnancy-related behavioral items”, we also suggest the need to identify alternative items capturing the conceptual domain of fertility-related behavior in settings where preconception and contraceptive care are low.

The Performance Monitoring for Action (PMA) Ethiopia survey, used in our study, covers several topics related to fertility, maternal, and newborn care. During the development of the questionnaire, we prioritized measures that had been extensively used and validated elsewhere, as we lacked sufficient resources to undertake the level of formative research necessary to develop and/or extensively modify measures. The established validity of the LMUP, as noted by the authors, motivated its inclusion in our survey. Based on our experience, we agree with the authors that researchers who plan to use the LMUP should engage in comprehensive cultural adaptation, especially when considering pregnancy-related behavioral items that need to reflect relevant practices of the populations under investigation.

Our objective was not to pursue an abbreviated version of the LMUP. Our recommendation to use such a meause was grounded in an effort to maximize the utility of survey items, including the majority of LMUP items that performed well, while supporting efficiency in future research by identifying items that performed poorly, as we recognize these are perpetual concerns in survey design. As for the placement of the LMUP items, unfortunately, we cannot test whether question ordering affected the validity of the scale. The pilot testing of the Performance Monitoring for Action instrument highlighted concerns about length and ordering of questions, relevant to both the LMUP and other questions, which resulted in adaptation to improve the overall survey flow. Whether these changes resulted in poorer validity cannot be tested but we acknowledge the authors’ concerns.

We thank the authors for this opportunity to engage in a productive conversation to move the measurement field forward. We look forward to connecting with the authors and the existing community of LMUP researchers to continue this important work.

## Declaration of Competing Interest

The authors declare the following financial interests/personal relationships which may be considered as potential competing interests: Celia Karp reports financial support was provided by Bill & Melinda Gates Foundation. Caroline Moreau reports financial support was provided by Bill & Melinda Gates Foundation. Solomon Shiferaw reports financial support was provided by Bill & Melinda Gates Foundation. Assefa Seme reports financial support was provided by Bill & Melinda Gates Foundation. Mahari Yihdego reports financial support was provided by Bill & Melinda Gates Foundation. Linnea Zimmerman reports financial support was provided by Bill & Melinda Gates Foundation.
